# Supplementary material for: Evaluation of Five Mammalian Models for Human Disease Research Using Genomic and Bioinformatic Approaches
Source: Biomedicines. 2023 Aug 4;11(8):2197. doi: 10.3390/biomedicines11082197 (PMC10452283; doi:10.3390/biomedicines11082197)
Supplement: Supplementary file 1 [file biomedicines-11-02197-s001.zip › Supplementary_Table_S1.pdf]

Supplementary Table S1. Details of the genome assembly information of the species.

Genome parameters	*Homo sapiens* (Human)	*Macaca mulatta* (Rhesus macaque)	*Callithrix jacchus* (Marmoset)	*Sus scrofa* (Pig)	*Mus musculus* (Mouse)	*Rattus norvegicus* (Rat)
**Coding genes**	19,962	21,591	22,252	21,280	21,848	22,250
**Non coding genes**	23,998	12,904	21,259	8,979	14,343	8,934
**Small non coding genes***	4,867	4,712	2,583	2,165	4,338	5,122
**Long non coding genes#**	16,90	4,773	7,174	6,797	9,969	3,288
**Misc. non coding genes**	2,221	3,419	104	17	36	524
**Pseudogenes**	15,229	767	11,398	1,626	13,638	1668
**Genome length**	3,096,649,726	2,971,331,530	2,897,824,427	2,501,912,388	2,728,222,451	2,870,184,193
**chromosomes**	1-22, X and Y	1-20, X and Y	1-22, X and Y	1-18, X and Y	1-21, X and Y	1-20, X and Y
**Assembly**	GCA_000001405.28	GCA_003339765.3	GCF_009663435.1	GCA_000003025.6	GCA_000001635.9	GCA_000001895.4
**Resource**	Ensembl	Ensembl	NCBI	Ensembl	Ensembl	Ensembl
**Gaps between scaffolds**	349	41	37	93	143	440
**Number of scaffolds**	472	2,979	993	706	102	1,395
**Scaffold N50**	67,794,873	82,346,004	98,198,953	88,231,837	106,145,001	14,986,627
**Scaffold L50**	16	14	12	9	11	65
**Number of contigs**	998	3,182	1,335	1,118	306	75,697
**Contig N50**	57,879,411	46,608,966	25,227,091	48,231,277	59,462,871	100,461
**Contig L50**	18	22	31	15	15	7,356
**Assembly level**	Chromosome	Chromosome	Chromosome	Chromosome	Chromosome	Chromosome

*Small non-coding genes - small non-coding genes with size < 200nt (e.g. miRNA, piRNA)

#Long non-coding genes (that produce transcripts of over >=200 nucleotides in length which do not appear to be translated) (e.g. lincRNA).
