# Supplementary material for: Evaluation of Five Mammalian Models for Human Disease Research Using Genomic and Bioinformatic Approaches
Source: Biomedicines. 2023 Aug 4;11(8):2197. doi: 10.3390/biomedicines11082197 (PMC10452283; doi:10.3390/biomedicines11082197)
Supplement: Supplementary file 1 [file biomedicines-11-02197-s001.zip › Supplementary_Table_S11.pdf]

Supplementary Table S11. Genes in each species that are identified with the highest number of human diseases in each human chromosome.

Chromosome	Rat*	Diseases	Mouse*	Diseases	Pig*	Diseases	Marmoset*	Diseases	Rhesus macaque*	Diseases
1	*MUTYH*	94	*MUTYH*	64	*MUTYH*	119	*MUTYH*	134	*MUTYH*	139
2	*MSH6*	227	*MSH6*	283	*MSH6*	382	*MSH6*	544	*MSH6*	588
3	*CTNNB1*	195	*VHL*	112	*VHL*	124	*VHL*	200	*VHL*	254
4	*WFS1*	48	*PDGFRA*	106	*PDGFRA*	108	*PDGFRA*	152	*PDGFRA*	141
5	*APC*	609	*APC*	634	*APC*	1680	*APC*	1394	*APC*	1551
6	*DSP*	100	*DSP*	84	*DSP*	163	*DSP*	148	*DSP*	153
7	*RELN*	23	*RELN*	26	*EGFR*	95	*EGFR*	177	*EGFR*	201
8	*NBN*	55	*PTK2B*	60	*ESRP1*	180	*ESRP1*	180	*ESRP1*	180
9	*SPTAN1*	239	*CDKN2A*	112	*NOTCH1*	164	*SPTAN1*	272	*SPTAN1*	277
10	*RET*	93	*RET*	105	*RET*	107	*RET*	173	*RET*	240
11	*PSMD13*	160	*PSMD13*	160	*PSMD13*	160	*MYO7A*	131	*MYO7A*	137
12	*POLE*	129	*POLE*	123	*POLE*	182	*POLE*	268	*POLE*	312
13	*RB1*	13	*RB1*	17	*RB1*	52	*RB1*	62	*RB1*	69
14	*DICER1*	65	*DYNC1H1*	44	*DYNC1H1*	84	*DYNC1H1*	131	*DYNC1H1*	107
15	*FBN1*	131	*FBN1*	108	*FBN1*	143	*FBN1*	273	*FBN1*	317
16	*TSC2*	358	*TSC2*	359	*TSC2*	609	*TSC2*	848	*TSC2*	885
17	*BRIP1*	96	*BRIP1*	98	*SCN4A*	180	*BRIP1*	213	*BRIP1*	227
18	*CDH20*	25	*SMAD4*	15	*CDH20*	28	*CDH20*	34	*LOXHD1*	35
19	*LDLR*	170	*LDLR*	229	*STK11*	320	*STK11*	477	*LDLR*	287
20	*ASXL1*	57	*ITPA*	47	*ASXL1*	192	*ASXL1*	254	*ASXL1*	274
21	*CBS*	18	*CBS*	26	*COL6A1*	44	*COL6A1*	44	*COL6A1*	50
22	*TMPRSS6*	66	*TMPRSS6*	66	*NF2*	109	*DEPDC5*	108	*NF2*	143
X	*FLNA*	52	*FLNA*	46	*FLNA*	138	*FLNA*	129	*FLNA*	183

* Genes associate with human diseases
