# Supplementary material for: Evaluation of Five Mammalian Models for Human Disease Research Using Genomic and Bioinformatic Approaches
Source: Biomedicines. 2023 Aug 4;11(8):2197. doi: 10.3390/biomedicines11082197 (PMC10452283; doi:10.3390/biomedicines11082197)
Supplement: Supplementary file 1 [file biomedicines-11-02197-s001.zip › Supplementary_Table_S2.pdf]

Supplementary Table S2. Distribution analysis of all CDS comparison between human and other species

	Rhesus macaque	Marmoset	Pig	Mouse	Rat
**Number of values**	17638	17787	14992	13806	13222
**Minimum**	71.74	71.63	70.81	70.11	68.93
**25% Percentile***	96.15	93.38	86.82	83.92	83.81
**Median**	97.29	95.29	89.89	86.99	86.88
**75% Percentile****	98.15	96.72	92.44	89.64	89.53
**Maximum**	100.0	100.0	100.0	100.0	100.0
**Range**	28.26	28.37	29.19	29.89	31.07
**Mean**	96.82	94.65	89.37	86.65	86.53
**Std. Deviation**	2.212	3.025	4.178	4.159	4.164
**Std. Error of Mean**	0.01666	0.02268	0.03412	0.03539	0.03621

* Number of CDS that were found at the 25% percentile of the distribution. ** Number of CDS that were found at the 25% percentile of the distribution.
