# Supplementary material for: Evaluation of Five Mammalian Models for Human Disease Research Using Genomic and Bioinformatic Approaches
Source: Biomedicines. 2023 Aug 4;11(8):2197. doi: 10.3390/biomedicines11082197 (PMC10452283; doi:10.3390/biomedicines11082197)
Supplement: Supplementary file 1 [file biomedicines-11-02197-s001.zip › Supplementary_Table_S5.pdf]

Supplementary Table S5. Distribution analysis of 10,316 commons CDS comparison between human and other species

	Rhesus macaque	Marmoset	Pig	Mouse	Rat
**Number of values**	10316	10316	10316	10316	10316
**Minimum**	73.32	74.47	72.54	70.11	71.73
**25% Percentile***	96.96	94.74	88.24	84.69	84.50
**Median**	97.75	96.05	90.73	87.46	87.32
**75% Percentile****	98.40	97.12	92.92	89.89	89.79
**Maximum**	100.0	100.0	99.67	98.98	100.0
**Range**	26.68	25.53	27.14	28.86	28.28
**Mean**	97.53	95.76	90.38	87.19	87.04
**Std. Deviation**	1.532	1.948	3.538	3.838	3.842
**Std. Error of Mean**	0.01508	0.01917	0.03483	0.03779	0.03782

* Number of CDS that were found at the 25% percentile of the distribution. ** Number of CDS that were found at the 25% percentile of the distribution.
