# Supplementary material for: Evaluation of Five Mammalian Models for Human Disease Research Using Genomic and Bioinformatic Approaches
Source: Biomedicines. 2023 Aug 4;11(8):2197. doi: 10.3390/biomedicines11082197 (PMC10452283; doi:10.3390/biomedicines11082197)
Supplement: Supplementary file 1 [file biomedicines-11-02197-s001.zip › Supplementary_Table_S6.pdf]

Supplementary Table S6. Human CDS identified across genome of different species.

Human CDS identified across the rhesus macaque genome.

		**Rhesus macaque chromosomes**																					
		**1**	**2**	**3**	**4**	**5**	**6**	**7**	**8**	**9**	**10**	**11**	**12**	**13**	**14**	**15**	**16**	**17**	**18**	**19**	**20**	**X**	**Y**
**Human chromosomes**	**1**	1799	-	3	2	-	1	4	-	1	-	1	-	1	1	1	-	-	-	1	1	1	-
	**2**	2	1	-	2	-	1	-	-	1	2	-	573	559	4	3	-	-	-	-	-	-	-
	**3**	-	988	1	-	1	1	-	-	-	-	-	1	-	-	-	-	1	-	-	1	2	-
	**4**	-	-	1	-	676	3	-	1	1	-	-	-	-	-	-	-	-	-	-	-	-	-
	**5**	-	1	-	-	1	761	1	-	-	1	-	-	1	-	-	-	-	2	-	-	-	-
	**6**	-	-	2	929	1	-	3	-	-	-	-	-	-	-	-	1	-	-	-	-	-	-
	**7**	1	1	808	-	-	-	1	1	-	-	2	-	-	-	1	1	-	-	2	-	-	-
	**8**	1	-	-	1	-	-	1	600	-	-	-	-	-	-	-	1	-	-	-	2	-	-
	**9**	1	1	1	-	-	-	-	-	-	-	1	-	-	-	687	-	-	-	-	-	-	-
	**10**	-	-	-	-	-	1	-	-	651	-	-	2	-	-	1	1	-	-	-	-	-	-
	**11**	6	-	1	-	-	-	-	2	-	1	-	-	-	1096	-	-	-	-	-	-	-	-
	**12**	1	-	-	3	-	-	1	-	1	-	929	-	-	1	-	1	-	-	1	1	1	-
	**13**	-	5	-	1	-	1	-	-	-	1	-	-	-	-	-	-	292	-	-	-	1	-
	**14**	-	-	-	2	-	-	554	-	-	-	1	1	-	-	-	-	-	-	-	-	-	-
	**15**	-	-	1	-	-	1	522	-	-	-	1	-	-	-	-	1	-	-	-	-	-	-
	**16**	-	1	-	2	-	1	1	-	1	-	1	-	-	-	-	-	-	-	-	719	1	-
	**17**	1	-	-	1	1	-	-	-	-	-	2	1	-	1	-	1019	-	-	-	-	-	-
	**18**	-	2	-	1	-	1	1	-	-	-	1	-	-	-	1	1	-	238	-	-	-	-
	**19**	-	1	1	-	-	-	1	-	-	-	1	-	-	-	-	2	-	-	1129	-	2	-
	**20**	1	-	-	-	1	-	-	-	-	479	-	-	1	-	-	-	-	-	-	-	1	-
	**21**	-	-	170	2	-	-	-	-	-	-	-	1	-	-	-	-	-	-	-	-	-	-
	**22**	-	1	3	1	-	1	-	-	-	384	-	-	-	-	-	-	-	-	-	-	-	1
	**X**	1	-	-	-	-	-	-	-	2	-	1	-	1	2	-	-	-	-	-	-	758	-
	**Y**	-	-	-	-	-	-	-	-	-	-	-	-	-	-	-	-	-	-	-	-	5	16

Human CDS identified across the marmoset genome

		Marmoset chromosomes																							
		**1**	**2**	**3**	**4**	**5**	**6**	**7**	**8**	**9**	**10**	**11**	**12**	**13**	**14**	**15**	**16**	**17**	**18**	**19**	**20**	**21**	**22**	**X**	**Y**
**Human chromosomes**	**1**	4	3	1	7	1	1	992	2	2	10	-	1	-	-	-	-	1	503	328	-	-	-	2	-
	**2**	5	1	1	4	3	573	3	1	-	-	3	1	-	554	1	-	1	-	-	-	-	-	-	-
	**3**	1	1	-	-	4	2	-	1	-	3	-	-	-	-	648	-	325	-	-	-	21	1	2	-
	**4**	3	1	680	-	-	1	-	-	-	-	-	1	21	-	1	1	-	-	-	-	-	1	2	-
	**5**	1	776	-	-	-	-	-	-	-	2	-	-	-	1	1	-	-	-	-	1	-	1	1	-
	**6**	1	1	-	947	2	2	-	1	-	1	-	1	-	-	-	-	-	-	-	-	-	1	-	-
	**7**	3	228	-	-	2	3	-	560	-	4	-	-	-	1	-	-	1	-	-	-	-	1	-	-
	**8**	-	1	-	2	2	1	1	-	-	1	-	2	240	-	1	383	-	-	-	-	-	-	-	-
	**9**	675	5	-	-	-	-	1	1	3	2	-	-	1	-	-	-	-	1	-	-	-	-	1	-
	**10**	-	1	-	-	1	2	138	-	-	-	-	532	-	1	-	-	-	-	-	-	-	-	-	-
	**11**	1	-	-	-	2	-	3	-	-	-	1142	-	1	-	-	4	-	-	-	-	-	-	-	-
	**12**	-	2	1	-	4	1	1	3	932	2	-	-	1	1	1	-	1	-	1	1	-	-	1	-
	**13**	157	-	-	-	131	1	-	-	1	-	-	-	-	-	-	1	-	1	-	-	3	-	1	-
	**14**	-	-	1	1	1	-	-	1	1	565	-	-	-	-	-	-	-	-	2	-	-	-	1	-
	**15**	-	-	-	-	1	142	1	-	3	386	-	-	-	-	-	-	-	-	-	-	-	-	-	-
	**16**	1	1	1	-	-	-	-	-	2	2	-	389	-	-	1	1	-	1	-	344	-	-	-	-
	**17**	6	1	-	1	1015	1	-	-	1	-	3	-	-	-	-	1	-	1	1	-	-	-	-	-
	**18**	1	1	-	1	1	-	-	-	-	1	2	-	239	-	-	-	-	-	-	-	-	-	-	-
	**19**	-	2	1	1	-	-	1	1	-	1	-	-	-	-	-	-	1	-	1	-	-	1102	1	-
	**20**	1	-	-	-	497	1	1	-	-	-	-	-	-	-	-	-	-	-	-	-	-	-	-	-
	**21**	-	-	-	2	1	-	-	-	-	1	-	-	-	1	-	-	-	-	-	1	176	-	-	-
	**22**	371	-	-	-	-	-	2	1	1	-	1	-	-	-	1	-	1	-	1	-	-	-	-	-
	**X**	4	-	1	1	1	-	2	-	1	-	-	3	-	-	-	-	-	-	-	-	-	1	706	3
	**Y**	1	-	-	-	4	-	-	-	-	-	-	-	-	-	-	-	-	-	-	-	-	-	6	15

Human CDS identified across the pig genome

		Pig chromosomes																			
		**1**	**2**	**3**	**4**	**5**	**6**	**7**	**8**	**9**	**10**	**11**	**12**	**13**	**14**	**15**	**16**	**17**	**18**	**X**	**Y**
**Human chromosomes**	**1**	1	26	2	518	1	667	17	-	182	111	-	1	-	48	-	-	-	7	1	-
	**2**	5	-	493	-	-	-	-	-	2	1	-	-	-	4	515	-	-	-	-	-
	**3**	-	1	2	-	-	-	4	-	1	-	-	1	885	-	-	-	-	-	-	-
	**4**	-	-	1	1	-	1	1	504	-	-	-	-	-	16	37	1	4	-	4	-
	**5**	-	393	1	-	-	1	1	-	1	-	-	-	1	2	1	289	-	-	1	-
	**6**	352	1	-	2	-	-	455	-	1	-	-	2	-	-	-	-	1	-	1	1
	**7**	2	1	186	-	-	-	1	1	181	-	-	1	1	1	-	-	-	299	-	-
	**8**	-	-	-	329	-	-	2	-	-	1	-	1	-	76	47	-	49	-	-	-
	**9**	429	1	15	-	1	-	-	-	-	66	-	-	-	15	-	-	-	1	1	-
	**10**	-	-	-	-	-	-	-	-	-	112	-	1	-	470	1	-	-	-	-	-
	**11**	-	484	-	-	-	-	-	-	430	-	-	-	-	-	-	-	1	-	-	-
	**12**	1	2	1	-	625	1	1	-	-	-	-	2	-	187	-	1	-	1	2	-
	**13**	-	1	-	1	1	-	-	-	-	-	254	-	-	2	-	-	-	-	1	-
	**14**	109	-	-	-	-	-	339	-	-	-	-	-	-	-	-	-	-	-	-	-
	**15**	311	-	-	1	1	-	178	-	-	-	-	-	-	-	4	1	-	-	-	-
	**16**	-	-	331	-	1	284	-	-	-	-	-	-	-	-	-	-	-	-	-	-
	**17**	3	1	-	1	1	-	-	-	-	-	-	870	-	-	-	-	-	-	1	-
	**18**	105	-	-	-	-	106	-	-	-	-	-	1	-	-	-	1	-	-	-	-
	**19**	-	391	-	-	-	451	-	-	-	-	-	-	-	-	-	-	-	-	1	-
	**20**	-	-	-	-	-	-	1	1	-	-	-	-	-	1	-	-	399	-	1	-
	**21**	-	-	-	-	-	-	2	-	-	-	-	-	125	-	-	-	-	-	-	-
	**22**	-	-	-	-	178	-	1	-	1	-	-	-	-	141	-	-	-	-	-	-
	**X**	-	-	2	1	1	-	1	-	-	-	-	3	1	2	-	2	-	-	536	6
	**Y**	-	-	-	-	-	-	-	-	-	-	-	-	-	-	-	-	-	-	3	14

Human CDS identified across the mouse genome

		Mouse chromosomes																					
		**1**	**2**	**3**	**4**	**5**	**6**	**7**	**8**	**9**	**10**	**11**	**12**	**13**	**14**	**15**	**16**	**17**	**18**	**19**	**20**	**21**	**X**	**Y**
**Human chromosomes**	**1**	387	4	399	556	24	6	1	30	1	1	25	-	18	2	-	1	1	1	-	-	-	1	-
	**2**	376	192	-	-	40	102	4	-	-	15	54	73	-	-	1	1	94	12	1	-	-	-	-
	**3**	-	-	108	-	-	132	-	-	274	-	-	-	2	82	-	227	10	-	-	-	-	-	-
	**4**	1	-	142	-	261	19	-	96	-	-	1	-	-	-	-	-	-	1	-	-	-	2	-
	**5**	6	1	-	-	-	1	-	-	-	-	133	-	238	-	68	2	17	168	1	-	-	1	-
	**6**	26	1	-	36	-	1	1	-	53	194	2	-	132	2	-	1	278	-	-	-	-	1	-
	**7**	-	1	-	1	269	242	-	-	10	2	41	59	22	-	1	-	-	-	1	-	-	-	-
	**8**	48	1	31	57	-	-	-	100	-	-	-	1	3	69	173	7	1	-	-	-	-	-	-
	**9**	1	206	-	202	-	1	-	-	-	-	-	-	60	-	-	-	1	-	63	-	-	1	-
	**10**	1	78	-	-	-	16	62	3	-	62	1	-	18	82	-	1	-	17	218	-	-	-	-
	**11**	-	143	-	5	-	-	291	-	230	-	-	1	-	-	1	-	-	-	222	-	-	-	-
	**12**	-	-	-	-	166	171	1	-	2	252	2	-	2	-	158	3	1	-	-	-	-	1	-
	**13**	5	-	18	-	33	1	-	41	-	-	-	-	-	150	2	1	-	1	-	-	-	2	-
	**14**	-	1	-	-	-	-	-	1	-	-	1	327	-	136	-	-	-	-	-	-	-	2	-
	**15**	-	143	1	-	-	1	119	-	197	-	-	-	-	6	-	-	1	1	-	-	-	-	-
	**16**	-	-	-	-	-	-	139	268	-	-	5	-	-	-	1	58	75	-	1	-	-	-	-
	**17**	-	-	-	-	-	1	-	-	-	1	839	-	-	-	1	-	1	-	-	-	-	1	-
	**18**	20	1	-	-	2	-	-	-	-	-	-	1	-	-	-	-	24	157	-	-	-	-	-
	**19**	-	1	-	-	-	-	356	121	44	88	-	-	4	-	-	-	54	-	-	-	-	1	-
	**20**	-	381	3	-	-	-	-	-	-	1	-	-	-	-	-	1	-	-	-	-	-	1	-
	**21**	-	-	-	-	-	-	-	1	-	29	-	-	2	-	-	80	12	-	-	-	-	-	-
	**22**	-	-	-	-	20	9	-	5	1	29	44	-	-	1	150	40	-	-	-	-	-	-	-
	**X**	1	-	-	1	1	-	1	-	-	-	4	2	-	1	-	2	1	-	1	-	-	480	-
	**Y**	-	-	-	-	-	-	-	-	-	-	-	-	-	-	-	-	-	1	-	-	-	8	1

Human CDS identified across the rat genome

		Rat chromosomes																					
		**1**	**2**	**3**	**4**	**5**	**6**	**7**	**8**	**9**	**10**	**11**	**12**	**13**	**14**	**15**	**16**	**17**	**18**	**19**	**20**	**X**	**Y**
**Human chromosomes**	**1**	2	390	4	6	538	-	1	-	-	18	-	-	364	28	1	-	17	-	31	1	1	-
	**2**	4	-	182	95	1	203	4	1	310	1	1	-	35	51	1	-	-	14	2	14	-	-
	**3**	1	109	-	118	1	-	2	259	7	1	219	1	-	-	27	51	-	-	-	-	-	-
	**4**	-	133	-	18	1	1	1	1	-	1	1	-	1	245	-	61	-	-	25	-	2	-
	**5**	31	225	1	1	-	-	1	-	13	134	2	1	-	-	-	1	50	153	-	-	3	-
	**6**	159	5	1	2	34	-	-	47	111	4	-	-	-	-	2	-	127	-	-	217	-	-
	**7**	-	-	1	323	-	58	2	9	1	3	-	151	-	37	-	-	23	-	-	1	1	-
	**8**	1	30	2	-	98	1	161	1	-	3	7	-	1	-	67	96	1	-	-	1	2	-
	**9**	59	-	195	2	194	-	1	-	1	-	-	-	-	-	-	2	49	-	-	-	1	-
	**10**	265	-	1	14	-	-	-	-	1	2	1	-	-	-	28	47	103	-	3	58	-	-
	**11**	502	2	125	-	-	3	-	213	1	-	1	-	-	-	1	-	-	-	-	-	-	-
	**12**	1	-	-	150	1	-	392	3	4	1	3	153	-	-	-	-	2	-	-	-	3	-
	**13**	-	14	1	1	-	-	2	-	5	1	-	29	-	-	132	42	-	1	-	-	2	-
	**14**	-	-	-	-	-	315	-	-	-	-	-	-	-	-	130	-	-	1	-	-	1	-
	**15**	115	-	132	-	1	-	-	198	-	-	-	-	-	-	6	-	-	-	-	-	-	-
	**16**	129	-	1	-	-	1	2	1	-	129	-	-	-	-	-	1	2	-	255	-	-	-
	**17**	-	1	1	-	-	1	1	-	-	826	-	1	-	-	-	1	-	-	-	1	2	-
	**18**	-	-	2	-	-	1	-	-	26	1	-	-	19	1	-	-	-	145	-	-	-	-
	**19**	348	1	1	-	-	-	96	39	30	1	-	16	-	-	-	62	1	-	40	-	1	-
	**20**	-	-	363	1	-	-	-	-	-	-	-	-	-	-	-	-	-	-	-	1	1	-
	**21**	-	-	-	-	-	-	-	-	-	-	77	-	-	-	-	-	2	-	-	41	-	-
	**22**	-	-	-	12	-	1	151	-	-	-	43	15	-	44	-	-	-	-	6	17	-	-
	**X**	6	3	-	1	1	1	-	5	1	5	-	2	1	2	-	1	2	1	-	2	424	-
	**Y**	-	-	-	-	-	-	-	-	-	-	-	-	-	-	-	-	-	1	-	-	7	1
