# Supplementary material for: Evaluation of Five Mammalian Models for Human Disease Research Using Genomic and Bioinformatic Approaches
Source: Biomedicines. 2023 Aug 4;11(8):2197. doi: 10.3390/biomedicines11082197 (PMC10452283; doi:10.3390/biomedicines11082197)
Supplement: Supplementary file 1 [file biomedicines-11-02197-s001.zip › Supplementary_Table_S7.pdf]

Supplementary Table S7. Conserved human CDS identified across genome of different species.

Identified 10316 conserved CDS mapped between human and rhesus macaque genome

		**Rhesus macaque chromosomes**																					
		**1**	**2**	**3**	**4**	**5**	**6**	**7**	**8**	**9**	**10**	**11**	**12**	**13**	**14**	**15**	**16**	**17**	**18**	**19**	**20**	**X**	**Y**
**Human chromosomes**	**1**	1072	-	-	1	-	1	4	-	1	-	1	-	1	-	-	-	-	-	-	-	-	-
	**2**	1	1	-	-	-	-	-	-	-	-	-	386	359	2	1	-	-	-	-	-	-	-
	**3**	-	642	1	-	1	-	-	-	-	-	-	1	-	-	-	-	-	-	-	-	-	-
	**4**	-	-	-	-	383	1	-	-	1	-	-	-	-	-	-	-	-	-	-	-	-	-
	**5**	-	1	-	-	1	495	1	-	-	1	-	-	1	-	-	-	-	1	-	-	-	-
	**6**	-	-	-	568	1	-	3	-	-	-	-	-	-	-	-	1	-	-	-	-	-	-
	**7**	-	1	463	-	-	-	1	1	-	-	1	-	-	-	-	1	-	-	2	-	-	-
	**8**	-	-	-	1	-	-	1	369	-	-	-	-	-	-	-	-	-	-	-	-	-	-
	**9**	-	1	1	-	-	-	-	-	-	-	1	-	-	-	396	-	-	-	-	-	-	-
	**10**	-	-	-	-	-	-	-	-	422	-	-	2	-	-	-	1	-	-	-	-	-	-
	**11**	-	-	1	-	-	-	-	-	-	1	-	-	-	615	-	-	-	-	-	-	-	-
	**12**	1	-	-	1	-	-	1	-	1	-	574	-	-	1	-	1	-	-	1	-	1	-
	**13**	-	-	-	1	-	1	-	-	-	1	-	-	-	-	-	-	178	-	-	-	1	-
	**14**	-	-	-	1	-	-	358	-	-	-	1	-	-	-	-	-	-	-	-	-	-	-
	**15**	-	-	1	-	-	-	368	-	-	-	-	-	-	-	-	1	-	-	-	-	-	-
	**16**	-	-	-	1	-	1	-	-	-	-	-	-	-	-	-	-	-	-	-	375	1	-
	**17**	-	-	-	-	1	-	-	-	-	-	1	1	-	-	-	632	-	-	-	-	-	-
	**18**	-	-	-	-	-	-	1	-	-	-	1	-	-	-	-	-	-	155	-	-	-	-
	**19**	-	1	-	-	-	-	1	-	-	-	1	-	-	-	-	-	-	-	446	-	2	-
	**20**	-	-	-	-	-	-	-	-	-	280	-	-	1	-	-	-	-	-	-	-	1	-
	**21**	-	-	74	2	-	-	-	-	-	-	-	-	-	-	-	-	-	-	-	-	-	-
	**22**	-	-	1	1	-	-	-	-	-	200	-	-	-	-	-	-	-	-	-	-	-	-
	**X**	1	-	-	-	-	-	-	-	2	-	1	-	1	2	-	-	-	-	-	-	374	-
	**Y**	-	-	-	-	-	-	-	-	-	-	-	-	-	-	-	-	-	-	-	-	1	6

Identified 10316 conserved CDS mapped between human and marmoset genome

		Marmoset chromosomes																							
		**1**	**2**	**3**	**4**	**5**	**6**	**7**	**8**	**9**	**10**	**11**	**12**	**13**	**14**	**15**	**16**	**17**	**18**	**19**	**20**	**21**	**22**	**X**	**Y**
**Human chromosomes**	**1**	-	-	1	5	1	1	596	-	1	5	-	1	-	-	-	-	-	280	196	-	-	-	1	-
	**2**	1	1	1	2	2	381	1	-	-	-	2	1	-	356	1	-	1	-	-	-	-	-	-	-
	**3**	1	1	-	-	-	1	-	-	-	1	-	-	-	-	415	-	208	-	-	-	17	-	1	-
	**4**	3	1	378	-	-	-	-	-	-	-	-	-	-	-	-	1	-	-	-	-	-	-	1	-
	**5**	-	497	-	-	-	-	-	-	-	2	-	-	-	1	1	-	-	-	-	-	-	-	1	-
	**6**	1	-	-	570	1	2	-	-	-	-	-	-	-	-	-	-	-	-	-	-	-	-	-	-
	**7**	1	119	-	-	-	1	-	347	-	-	-	-	-	-	-	-	1	-	-	-	-	1	-	-
	**8**	-	-	-	1	1	-	1	-	-	1	-	-	127	-	1	240	-	-	-	-	-	-	-	-
	**9**	399	-	-	-	-	-	-	1	2	-	-	-	-	-	-	-	-	-	-	-	-	-	-	-
	**10**	-	-	-	-	1	1	84	-	-	-	-	344	-	-	-	-	-	-	-	-	-	-	-	-
	**11**	-	-	-	-	1	-	-	-	-	-	616	-	-	-	-	-	-	-	-	-	-	-	-	-
	**12**	-	2	-	-	1	-	-	2	575	-	-	-	-	-	1	-	-	-	-	-	-	-	1	-
	**13**	98	-	-	-	80	1	-	-	-	-	-	-	-	-	-	1	-	1	-	-	-	-	1	-
	**14**	-	-	1	1	1	-	-	1	-	355	-	-	-	-	-	-	-	-	-	-	-	-	1	-
	**15**	-	-	-	-	1	95	1	-	2	271	-	-	-	-	-	-	-	-	-	-	-	-	-	-
	**16**	1	-	1	-	-	-	-	-	1	-	-	188	-	-	1	-	-	-	-	186	-	-	-	-
	**17**	2	-	-	1	630	1	-	-	-	-	1	-	-	-	-	1	-	1	-	-	-	-	-	-
	**18**	-	-	-	-	-	-	-	-	-	1	1	-	155	-	-	-	-	-	-	-	-	-	-	-
	**19**	-	1	-	1	-	-	-	-	-	1	-	-	-	-	-	-	-	-	-	-	-	449	1	-
	**20**	1	-	-	-	281	-	-	-	-	-	-	-	-	-	-	-	-	-	-	-	-	-	-	-
	**21**	-	-	-	2	-	-	-	-	-	-	-	-	-	-	-	-	-	-	-	-	74	-	-	-
	**22**	199	-	-	-	-	-	1	-	-	-	-	-	-	-	-	-	1	-	-	-	-	-	-	-
	**X**	1	-	1	1	-	-	-	-	1	-	-	2	-	-	-	-	-	-	-	-	-	-	374	-
	**Y**	-	-	-	-	-	-	-	-	-	-	-	-	-	-	-	-	-	-	-	-	-	-	4	3

Identified 10316 conserved CDS mapped between human and pig genome

		Pig chromosomes																			
		**1**	**2**	**3**	**4**	**5**	**6**	**7**	**8**	**9**	**10**	**11**	**12**	**13**	**14**	**15**	**16**	**17**	**18**	**X**	**Y**
**Human chromosomes**	**1**	-	11	2	358	1	447	10	-	132	87	-	1	-	33	-	-	-	1	1	-
	**2**	2	-	360	-	-	-	-	-	2	-	-	-	-	2	384	-	-	-	-	-
	**3**	-	1	-	-	-	-	2	-	-	-	-	1	640	-	-	-	-	-	-	-
	**4**	-	-	-	1	-	-	-	337	-	-	-	-	-	13	27	1	3	-	4	-
	**5**	-	284	1	-	-	-	1	-	1	-	-	-	1	1	1	211	-	-	1	-
	**6**	258	1	-	2	-	-	305	-	-	-	-	2	-	-	-	-	-	-	1	1
	**7**	-	1	118	-	-	-	1	1	137	-	-	-	1	1	-	-	-	210	-	-
	**8**	-	-	-	243	-	-	2	-	-	-	-	-	-	56	33	-	34	-	-	-
	**9**	298	-	10	-	1	-	-	-	-	43	-	-	-	13	-	-	-	1	-	-
	**10**	-	-	-	-	-	-	-	-	-	84	-	1	-	346	1	-	-	-	-	-
	**11**	-	333	-	-	-	-	-	-	282	-	-	-	-	-	-	-	1	-	-	-
	**12**	1	1	1	-	442	-	1	-	-	-	-	2	-	127	-	-	-	-	2	-
	**13**	-	1	-	1	1	-	-	-	-	-	177	-	-	1	-	-	-	-	1	-
	**14**	80	-	-	-	-	-	247	-	-	-	-	-	-	-	-	-	-	-	-	-
	**15**	219	-	-	1	-	-	141	-	-	-	-	-	-	-	4	1	-	-	-	-
	**16**	-	-	186	-	1	188	-	-	-	-	-	-	-	-	-	-	-	-	-	-
	**17**	-	-	-	-	-	-	-	-	-	-	-	634	-	-	-	-	-	-	1	-
	**18**	83	-	-	-	-	74	-	-	-	-	-	-	-	-	-	-	-	-	-	-
	**19**	-	197	-	-	-	258	-	-	-	-	-	-	-	-	-	-	-	-	1	-
	**20**	-	-	-	-	-	-	1	1	-	-	-	-	-	1	-	-	278	-	1	-
	**21**	-	-	-	-	-	-	2	-	-	-	-	-	70	-	-	-	-	-	-	-
	**22**	-	-	-	-	116	-	-	-	-	-	-	-	-	81	-	-	-	-	-	-
	**X**	-	-	2	-	1	-	-	-	-	-	-	-	-	2	-	2	-	-	371	3
	**Y**	-	-	-	-	-	-	-	-	-	-	-	-	-	-	-	-	-	-	2	5

Identified 10316 conserved CDS mapped between human and mouse genome

		**Mouse chromosomes**																						
		**1**	**2**	**3**	**4**	**5**	**6**	**7**	**8**	**9**	**10**	**11**	**12**	**13**	**14**	**15**	**16**	**17**	**18**	**19**	**20**	**21**	**X**	**Y**
**Human chromosomes**	**1**	292	3	303	413	18	5	-	23	1	1	14	-	12	1	-	-	-	1	-	-	-	1	-
	**2**	278	146	-	-	30	85	3	-	-	12	51	58	-	-	-	-	75	11	1	-	-	-	-
	**3**	-	-	92	-	-	92	-	-	211	-	-	-	2	66	-	176	6	-	-	-	-	-	-
	**4**	1	-	106	-	186	17	-	73	-	-	1	-	-	-	-	-	-	-	-	-	-	2	-
	**5**	5	1	-	-	-	1	-	-	-	-	111	-	196	-	52	2	13	119	1	-	-	1	-
	**6**	22	-	-	31	-	-	1	-	45	161	1	-	103	1	-	1	207	-	-	-	-	1	-
	**7**	-	1	-	-	197	170	-	-	7	2	27	45	19	-	1	-	-	-	1	-	-	-	-
	**8**	39	1	21	48	-	-	-	75	-	-	-	-	1	52	129	5	1	-	-	-	-	-	-
	**9**	1	149	-	157	-	1	-	-	-	-	-	-	41	-	-	-	1	-	52	-	-	-	-
	**10**	1	57	-	-	-	11	41	3	-	47	1	-	10	68	-	1	-	13	179	-	-	-	-
	**11**	-	94	-	-	-	-	203	-	168	-	-	-	-	-	1	-	-	-	153	-	-	-	-
	**12**	-	-	-	-	127	122	1	-	1	203	1	-	2	-	120	3	1	-	-	-	-	1	-
	**13**	5	-	13	-	25	1	-	27	-	-	-	-	-	107	2	-	-	1	-	-	-	1	-
	**14**	-	-	-	-	-	-	-	1	-	-	-	258	-	99	-	-	-	-	-	-	-	2	-
	**15**	-	102	1	-	-	-	91	-	170	-	-	-	-	6	-	-	-	1	-	-	-	-	-
	**16**	-	-	-	-	-	-	96	186	-	-	3	-	-	-	1	47	44	-	1	-	-	-	-
	**17**	-	-	-	-	-	1	-	-	-	-	634	-	-	-	-	-	1	-	-	-	-	1	-
	**18**	17	-	-	-	1	-	-	-	-	-	-	1	-	-	-	-	18	120	-	-	-	-	-
	**19**	-	1	-	-	-	-	254	87	32	47	-	-	2	-	-	-	33	-	-	-	-	1	-
	**20**	-	280	-	-	-	-	-	-	-	1	-	-	-	-	-	-	-	-	-	-	-	1	-
	**21**	-	-	-	-	-	-	-	-	-	15	-	-	2	-	-	51	8	-	-	-	-	-	-
	**22**	-	-	-	-	16	4	-	5	-	13	30	-	-	-	107	27	-	-	-	-	-	-	-
	**X**	1	-	-	-	1	-	1	-	-	-	4	2	-	1	-	1	1	-	1	-	-	368	-
	**Y**	-	-	-	-	-	-	-	-	-	-	-	-	-	-	-	-	-	1	-	-	-	5	1

Identified 10316 conserved CDS mapped between human and rat genome

		Rat chromosomes																					
		**1**	**2**	**3**	**4**	**5**	**6**	**7**	**8**	**9**	**10**	**11**	**12**	**13**	**14**	**15**	**16**	**17**	**18**	**19**	**20**	**X**	**Y**
**Human chromosomes**	**1**	1	302	4	5	415	-	1	-	-	13	-	-	290	19	1	-	12	-	23	-	1	-
	**2**	4	-	146	83	1	160	3	-	251	-	-	-	29	50	-	-	-	11	-	12	-	-
	**3**	-	94	-	92	-	-	1	210	6	1	175	-	-	-	22	43	-	-	-	-	-	-
	**4**	-	106	-	17	1	-	1	1	-	1	1	-	1	184	-	49	-	-	22	-	2	-
	**5**	26	183	1	1	-	-	1	-	11	114	2	-	-	-	-	1	42	115	-	-	3	-
	**6**	127	4	-	-	30	-	-	41	95	3	-	-	-	-	1	-	99	-	-	174	-	-
	**7**	-	-	1	245	-	45	2	6	1	2	-	117	-	27	-	-	19	-	-	1	1	-
	**8**	-	21	1	-	86	1	127	1	-	1	5	-	1	-	52	74	1	-	-	1	-	-
	**9**	51	-	150	2	157	-	1	-	1	-	-	-	-	-	-	2	38	-	-	-	-	-
	**10**	219	-	-	11	-	-	-	-	1	1	1	-	-	-	27	41	80	-	3	47	-	-
	**11**	353	1	93	-	-	2	-	164	1	-	1	-	-	-	1	-	-	-	-	-	-	-
	**12**	1	-	-	119	1	-	322	1	4	1	3	125	-	-	-	-	2	-	-	-	2	-
	**13**	-	13	1	1	-	-	2	-	5	1	-	24	-	-	106	27	-	1	-	-	1	-
	**14**	-	-	-	-	-	258	-	-	-	-	-	-	-	-	100	-	-	1	-	-	1	-
	**15**	90	-	104	-	-	-	-	171	-	-	-	-	-	-	6	-	-	-	-	-	-	-
	**16**	97	-	-	-	-	-	2	-	-	93	-	-	-	-	-	1	-	-	184	-	-	-
	**17**	-	1	1	-	-	1	1	-	-	628	-	-	-	-	-	-	-	-	-	-	1	-
	**18**	-	-	1	-	-	1	-	-	19	-	-	-	17	1	-	-	-	117	-	-	-	-
	**19**	257	1	1	-	-	-	59	31	19	-	-	13	-	-	-	46	1	-	28	-	1	-
	**20**	-	-	279	1	-	-	-	-	-	-	-	-	-	-	-	-	-	-	-	1	1	-
	**21**	-	-	-	-	-	-	-	-	-	-	51	-	-	-	-	-	2	-	-	23	-	-
	**22**	-	-	-	4	-	1	112	-	-	-	26	12	-	34	-	-	-	-	5	6	-	-
	**X**	6	3	-	-	-	1	-	2	1	5	-	1	1	2	-	1	2	1	-	-	354	-
	**Y**	-	-	-	-	-	-	-	-	-	-	-	-	-	-	-	-	-	1	-	-	5	1
