# Supplementary figures and images for: Evaluation of Five Mammalian Models for Human Disease Research Using Genomic and Bioinformatic Approaches
Source: Biomedicines. 2023 Aug 4;11(8):2197. doi: 10.3390/biomedicines11082197 (PMC10452283; doi:10.3390/biomedicines11082197)

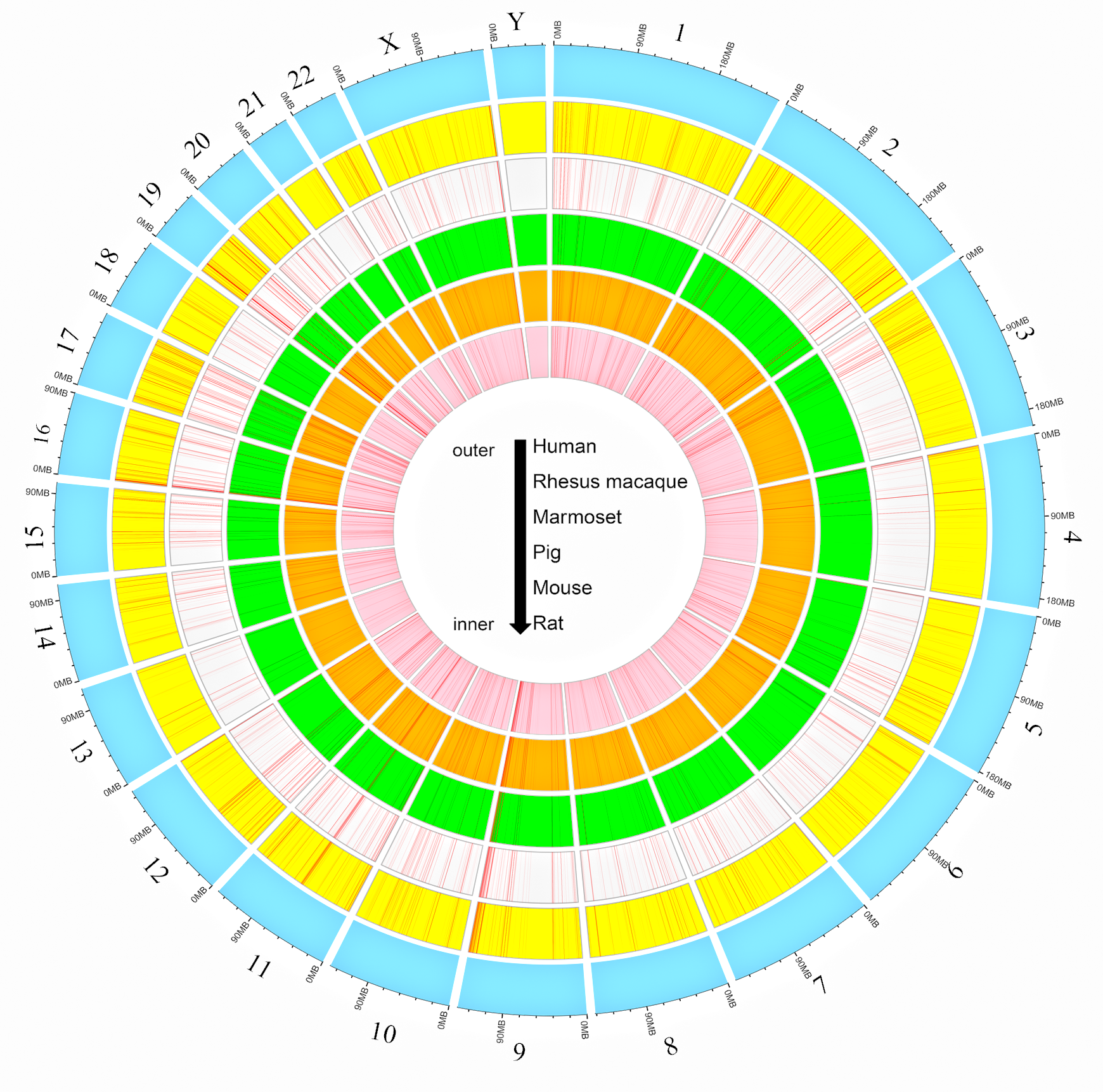

Supplement: Supplementary file 1 [file biomedicines-11-02197-s001.zip › Supplementary_Figure_S1.tif]
